# Lister: An Appreciation

**Published:** 1912-03

**Authors:** Robert Roxburgh


Vol. XXX. No. 115.

### The Bristol Medico-Chirurgical Journal.

*"Scire est nescire, nisi id me*
*Scire alius sciret."*

MARCH, 1912.

### LISTER:

AN APPRECIATION BY

Robert Roxburgh, M.B., F.R.C.S. Ed.

While the civilised world is mourning one of the supreme benefactors of the human race, who has passed away in a ripe and honoured old age, it may interest the readers of the *Journal*, especially its younger subscribers, to peruse some personal reminiscences of the man and his work by one who knew him intimately, and who worked under him in the later seventies, when he was still almost alone in his convictions and practice, the apostle of a new creed.

The present generation of young practitioners and students can form no adequate conception of surgical procedure or of the general aspects of surgical practice in pre-Listerian days. Cleansing of the skin, the hands and the instruments, now so obviously indispensable before an operation, so simple and reasonable and necessary, would forty years ago have been generally regarded as a meaningless precaution, while the boiling of instruments would have excited amazement and ridicule. It was a common practice for an operating surgeon to keep an old coat in the theatre, bespattered with blood and pus, which he donned for his operations, so as to protect his ordinary garments from being soiled. Inflammation, suppuration, pyæmia, surgical emphysema, erysipelas, hospital gangrene—these, in more or less acute form and variety, were regarded as normal sequences of wounds, whether inflicted by accident or by the surgeon. True, such complications were less liable to appear in private than in hospital work, and much less common where ordinary cleanliness with soap and water was observed, and frequently changed water-dressings were used. Healing by first intention also was by no means unknown, for Sir Astley Cooper's admirable advice was not lost on intelligent operators—to keep substantial flaps in *close apposition*, and to avoid pockets and fissures. But with every known care and precaution the issue of an operation was always most uncertain. It was the beginning of a voyage on an uncharted sea, and it was many days before the surgeon could, without dread, steer his patient into a safe harbour. Still more precarious was the issue of an accidental wound. The general mortality of compound fractures in England was about 33 per cent. Injuries to joints generally required immediate amputation, and those of the peritoneum were practically always fatal.

My first experience of surgery was in the surgical wards of the Glasgow Royal Infirmary a few years after Lister had been transferred from there to the Chair of Clinical Surgery in Edinburgh University. The impression his great discovery had made in the very hospital where the "germ theory" had become the basis of a great new departure may be gauged by the condition of these wards. The smell on entering was foul in the extreme. Most of the patients were suffering from fever, dry tongue, restlessness, and great pain. In one bed lay a man with a terrible lacerated wound from a circular saw. It was being treated by linseed poultices. Pus was oozing from the inflamed and swollen limb, the lymphatics of the arm were red and tender, and secondary abscesses were forming in the axilla. In another bed was a case of acute surgical emphysema of the scrotum following an operation, and so on. These experiences determined me to continue my studies in Edinburgh under Mr. Lister, whom I afterwards served as dresser, clerk and house-surgeon. The contrast there was extraordinary. Lister's wards were fresh and sweet. The wholesome odour of antiseptics was present, but never the sickly one of pus, or the horrid effluvia of putrefaction. Pyrexia, if it existed at all, was merely an occasional incident, and there was the same absence of acute suffering to which we are accustomed in modern days.

The clinical teaching in the lecture theatre was also of a novel kind. A few, a very few, "practical" students complained that instead of "tips" and useful details (all of which they could learn in the wards), they had to listen to frequent profound disquisitions on physiological and pathological truths, often illustrated by accounts of original experimental research. To students in a hurry the bearing of these facts and researches on their particular work, and especially their forthcoming examinations, was not immediately clear! On the other hand, the vast majority of men who thronged these benches, expectant and alert, fully recognised that what they listened to was of far-reaching significance, and that they were being grounded on the bed rock of scientific discovery. The style of the lectures, too—their pure, lucid, admirably selected English—was no inconsiderable element in their charm.

One has merely to glance at the list of papers on vitally important subjects which Lister had already published since his graduation as M.B. Lond. in 1852, enumerated in the *British Medical Journal* for February 17th, pp. 400, 401, to realise something of the quality of these lectures. In truth, it was the *principles* lying at the foundation of sound practice that were the professor's constant theme. Moreover, the new experience under antiseptic treatment had shed a flood of light on the reparative processes in the various tissues—processes identical in open wounds with those taking place under an unbroken skin—while the hoary fallacies of " laudable pus," " benign inflammation " in surface incisions, " healthy exudations," etc., were exposed. Again, the extreme value of free drainage in certain cases, the distinction between " bare " and " dead " bone, the functions of granulation tissue as a provisional constructive covering, whose surface was secretory but not absorbent, the mechanism of counter-irritation, the method of securing a " bloodless operation " on a limb by simply raising it into the vertical position for a few minutes till the veins were empty and the arteries contracted in response to the lowering of the *vis a fronte,* when a sufficiently strong elastic ligature immediately applied at the requisite upper limit produced the bloodless condition desired.

Such were a few of the topics dwelt on, their practical application being daily seen in the wards. Even the most pragmatical student could not fail to be interested when, in opposition to all the text-books of the day, he saw the Master without a moment's hesitation cut into a sound joint and remove loose cartilages which were causing inconvenience, or treat that puzzling affection, gelatinous degeneration or " white swelling " of the knee-joint (not then, of course, recognised as tubercular), with notable prescience by free incision, drainage of the synovial sac, and rest.

Another phenomenon not previously known, but now clearly demonstrated, was the healing of a deep wound, such as that of a compound fracture, by the gradual " organisation " of blood clot, the young epithelium spreading from the skin edges and meeting over the surface of the clot without the intervention of granulation tissue. [1]

[1] A beautiful example of this process in non-traumatic conditions came under the writer's notice in practice. A diabetic gentleman was threatened with gangrene of one foot. The anterior tibial artery was found to be thrombosed in nearly its whole extent. The symptoms passed away, and the foot recovered. Four months afterwards at the autopsy

But let it not be for a moment supposed that Lister's teaching was in the least degree defective on the practical side. No more ingenious master of surgical handicraft, no sounder practitioner as such, was to be found. He was a bold and purposeful operator, but care and delicacy of detail rather than speed and "dash" marked his operations. The well-known abdominal tourniquet, the conical bulb-ended urethral sounds (part of the armamentarium of every modern surgeon), the beautifully-designed splint for the after treatment of his wrist-joint excision, were developments arising in the course of a most comprehensive surgical experience. There may be mentioned here the button sutures, or, as he loved to call them, "artificial finger-tips," which proved invaluable in relieving the tension on approximating sutures in cases where large amounts of cutaneous and muscular tissue had had to be removed. A double row of small leaden plates, about three-quarters of an inch in length by half an inch in breadth and perforated in the centre, were placed at a considerable distance from the incision, and were united by very deep silver wires carried through the substance of the neighbouring parts and twisted round the buttons, thus bringing the divided tissues together *en masse*.

On the subject of anæsthetics, Lister's article in Holmes's *System of Surgery* was for long the last word on that subject, at least as regards chloroform, the great principles being *free access of air* and careful watching of the breathing for possible mechanical obstruction. But we heard him declare not long after that the article was defective in so far as it laid insufficient stress on the toxic action of a powerful drug on the nerve centres, especially on the respiratory centre in the medulla. Those who imbibed Lister's teaching on chloroform administration gained thereby a possession which never could fail them in after life.

the whole thrombus was found to be organised and vascularised, and one considerable arteriole ran through its entire length. Needless to say, such an event would be impossible under any but aseptic conditions, but it was not uncommon as a visible reality in Lister's wards.

Though it may seem superfluous in these days, we venture to remind the younger generation that next to the epoch-making discovery of the causes of sepsis and the means of its prevention stands the introduction of the catgut ligature, sufficient of itself to render the name of Lister immortal. Is it necessary to remind men of the present day that previous to his time arteries were tied with long silk ligatures, which were allowed to hang out of the corner of the wound, and were pulled away one by one by the surgeon when he believed he could do so with safety ?

As an accomplished chemist Lister was tireless in improving his antiseptic methods, in trying new germicides, in simplifying procedure. He adhered strictly to the use of the much derided spray in operating and dressing, till advancing bacteriological knowledge had fully convinced him that organisms floating in the air were so attenuated as to be a *quantité négligeable,* while the source of actual danger was *inoculation* from skin, hands, sponges, instruments, etc.

When Lister, to the poignant regret of the whole Edinburgh School and all sections of society there, both rich and poor, accepted the invitation to succeed Sir William Fergusson as Professor at King's College, London, he still regarded the spray as essential. He had adopted a steam spray-producer, and the old " donkey engine " of his dressers—now in the writer's possession—had been discarded for the more efficient apparatus. A very few years afterwards the spray was finally abandoned. At that date, 1877, the dressing of an aseptic wound consisted first in bathing the surface with 1 in 40 carbolic solution, then a little strip of " protective " (green oil-silk which had previously been brushed over with a weak solution of phenol in dextrine to enable it to take a uniformly wet surface, and kept dry for use in a tin box) was dipped in the lotion and laid on the incision or the granulating wound, cut just large enough to *protect* the growing epithelium from the gauze dressing and its inherent carbolic acid. Over the surface was laid a thin wet layer of carbolised gauze, and then eight layers of dry gauze, the latter consisting of sheets of gauze which had been dipped in a stable solution of phenol, in a mixture of resin and paraffin, dried and stored. Over all was a sheet of impervious jaconet, and finally the bandage, sometimes re-inforced at the edges with an elastic webbing. Such a dressing could be safely left undisturbed from four to six days. Complex as the description may sound, in hands familiar with it the dressing was simple. By and by other and easier methods were evolved till *antiseptic* surgery had, in all but traumatic cases, become still further idealised into *aseptic* surgery.

At the time of which we write Lister stood absolutely alone among his contemporaries as the founder and elucidator of a world-shaking reform in surgical theory and practice. He had written many articles and delivered addresses sufficient to enlighten all who cared to be illuminated. But the impression made was simply trifling except among the Assistant Surgeons and other men of less stereotyped views. His hopes were all set on the young. It was they who saw before their eyes things never before seen. They followed him in crowds round his wards. They listened to his simple doctrines, and they could not fail not only to be convinced, but themselves to become active and warm disciples.

The strange want of appreciation on the part of his own colleagues was undoubtedly largely the product of a jealous conservatism and largely, no doubt, was due to the etiquette, or whatever else we may call it, which disallowed the visits of these men to wards where abundant cases far more eloquent than words provided absolute proof of the truth of the new doctrines.

But while the prophet was still without honour in his own country, young graduates were carrying the truth through the land and to the colonies, and delegates from such foreign schools as Copenhagen, Paris, Munich, Vienna, Rome frequently arrived, sat at the Master's feet, saw his practice, listened to his teaching, and returned to their homes filled with an over-mastering enthusiasm. The case of Munich was exceptionally remarkable, but space forbids us to enlarge upon it. Those who would study the whole course of events will find it in Lister's own

Presidential Address at the British Association, Liverpool Meeting, in 1896, published in full in *Nature*.

When Lister took up his duties at King's College Hospital he could not count on a single convert to his views among men of his own professional status in the Metropolis, while even the younger surgeons of that day knew very little of the subject. And yet at that very time his name in many parts of Europe was regarded with veneration as that of one of the great reformers of history, as, for example, in Rome, where a statuette of the Master had been placed over the entrance of the new *Policlinica*. He had wisely taken with him to London such stalwarts as W. Watson Cheyne, and John Stewart now of Halifax, Nova Scotia, who saw to the interests of his patients and educated the nurses, students, and even members of the staff with sometimes amusing, sometimes less jocular, results. It was characteristic of the man that, fearing lest several chronic patients in his Edinburgh wards, mostly spinal and hip cases, might be discharged uncured, he had them transferred to a good private lodging, and there maintained at his own expense and carefully attended by his private assistant, Dr. Bishop, until they could be returned healed to their homes in different parts of Scotland.

Happily for mankind he had inherited a fortune which rendered him largely independent of private practice, and the greater part of his time was usually spent in his wards and in his private laboratory.

This brings us to an important element of Lister's enormous influence on the young and impressionable, which has received far too little notice in any of the recently-published obituaries. We refer to the moral qualities of the man and his teaching. The spell which he exercised on many minds was due to a commingling in his personality of the pure white light of exact science to which his intellect was devoted with a most ardent humanity and sympathy with suffering. His touch was delicate as a woman's, and his usually placid brow was seldom darkened except by some careless and rough handling of a patient. In these wards conscience underlay every act. No sloveniiness was allowed to pass muster, and even the insertion of a pin in a dressing was the outcome of the scientific conscience, as it was done in such manner as to secure the maximum of efficiency.

The wards were a school not only of the healing art, but of systematic kindness. His patients were not cases merely, but men, women and children, to whom, as members of the human family, full respect and consideration were due. Again, while he was the target of frequent misrepresentation, and even contemptuous allusion by men who should have known better, his chivalry and courtesy remained untarnished, and he treated his opponents with a generosity and an amiable tolerance which used to astonish his students. To the latter he was, above all men, the knight *sans peur et sans reproche,* and they rejoiced in that hero-worship which close contact with him inspired.

One thing which roused him to something like fury was quackery in any form. All unreality, pretentiousness, self-advertisement, claims to exceptional wisdom and knowledge, were to him anathema. He would often sadly declare that the worst quacks were those inside the boundaries of the medical profession. But he regarded those unscrupulous tricksters who filled their purses at the expense of credulous patients during long courses of absolutely worthless treatment as almost outside the pale of humanity.

In middle life Lister was a man of active and agile figure. He was a tennis-player and a rider. His face was not so much handsome as genuinely beautiful, for the features were delicately chiselled, the mouth very firm but very mobile, and often relaxing into a peculiarly charming smile, and the eyes thoughtful, penetrating and expressive. In his last years he allowed his beard and moustache to grow, and his face thereby became so altered as to be hardly recognisable except by those who had watched the change. Like the greatest men in science, the Newtons and the Kelvins, Lister was truly modest. Personal vanity or self-consciousness he did not seem to possess, and though virile and powerful in speech and action, he was, as his colleague, Professor Blackie, once said to the writer, a "virgin-minded man."

His work was done in deadly earnest, but not infrequently he would condescend to a little friendly chaff with his dressers, or with boyish merriment talk about "antisceptics," or in allusion to his preference for chloroform over ether, would laughingly quote the couplet :—

"How happy could I be with *either*,
Were t'other dear charmer away!"

The multitudinous honours which afterwards were showered upon this great man were never allowed to alter in the slightest degree his simple, kindly manners and warm interest in his old friends and pupils. To the writer, as he joined in the throng of mourners who attended the deeply impressive funeral service at Westminster Abbey, the dominant thought was the triumph of truth and character over indifference, hostility, time and distance. Thirty-five years ago this man, fired with love of scientific truth and love of his kind, was calling to deaf ears. To-day every scientific body, every University, almost every Government, bow their heads in silent recognition and profound mourning.

The great sorrow of his life came in 1893, when he lost the dearly-loved partner of his life. Lady Lister, a daughter of Professor Syme, had been from the first the support of his labours, the light of his home, the comrade of his fighting days, and his one and only assistant in all laboratory work. He was desolated by the loss, but his high Christian hopes and deep religious faith made him conqueror here as he had already been in relation to science and humanity.

